# Unveiling the Role of Mg^2+^ and Mn^2+^ in Theaflavin-Mediated Inhibition of Advanced Glycation End Products Formation: Mechanistic Insights from Intermolecular Interaction

**DOI:** 10.3390/molecules31152648

**Published:** 2026-07-29

**Authors:** Lixia Yuan, Yanqing Zhang, Huimin Liu, Xiaowei Lu, Yue Wang, Zhining Yan, Min Liu

**Affiliations:** 1 Shandong Key Laboratory of Applied Technology for Protein and Peptide Drugs, Institute of BioPharmaceutical Research, Liaocheng University, Liaocheng 252059, China; yuan1072165059@163.com (L.Y.); 17563586691@163.com (H.L.); m17863607367@163.com (X.L.); 18235664829@163.com (Z.Y.); 2 School of Chemistry and Chemical Engineering, Liaocheng University, Liaocheng 252059, China; zyq1281606004@163.com (Y.Z.); 18235782278@163.com (Y.W.)

**Keywords:** intermolecular interaction, human serum albumin (HSA), advanced glycation end products (AGEs), theaflavin (TA), metal ions, spectroscopic techniques

## Abstract

Advanced glycation end products (AGEs) are implicated in the pathogenesis of chronic diseases. This study investigated the inhibitory effect of theaflavin (TA) on AGE formation in a human serum albumin (HSA)-fructose model system, both in the absence and presence of non-cytotoxic Mg^2+^ or Mn^2+^. The underlying mechanism was elucidated using multiple spectroscopic techniques and molecular docking. TA significantly inhibited AGE formation, with the enhancement by metal ions following the order: 1.5 μM Mn^2+^ > 1.5 μM Mg^2+^ > 1 mM Mg^2+^. This trend aligned with the binding affinity between HSA and TA derived from molecular interaction studies. Site marker competition and docking results revealed that TA binds preferentially within subdomain IIA of HSA. Furthermore, the conformational changes in HSA following glycation and inhibition were monitored, along with the influence of metal ions on the antioxidant activity of TA. By integrating the results, it was concluded that free radical scavenging contributes more significantly to AGE inhibition than does blocking the glycation sites on HSA. Overall, this study elucidates the influence of metal ions on the inhibitory effect of TA against AGE formation and the corresponding mechanism, providing insights for the prevention and management of chronic diseases.

## 1. Introduction

Chronic diseases, including cardiovascular diseases, kidney diseases, diabetes, and cancers, account for over 70% of global mortality, as reported by the World Health Organization [1]. The pathogenesis of these diseases is closely linked to the accumulation of advanced glycation end products (AGEs), formed non-enzymatically via the Maillard reaction between reducing sugars and macromolecules such as proteins, nucleic acids, and lipids [2]. Given the significant health burden attributed to AGEs, the development of effective strategies to inhibit their formation is crucial for mitigating the progression of chronic diseases.

To reduce AGE levels, several approaches have been explored, including the use of pharmaceutical agents [3,4] and soluble receptors for AGEs [5]. However, these interventions are often limited by potential safety risks and high costs. Consequently, natural antioxidants have garnered significant interest as promising alternatives, owing to their potent anti-AGE activity and favorable safety profiles. For instance, Song et al. [6] demonstrated that ascorbic acid effectively suppresses AGE formation in a glucose-lysine model. Similarly, Ling et al. [7] reported that flavonols with varying *B*-ring structures inhibit AGE formation in a bovine serum albumin (BSA)-methylglyoxal system.

Tea, a widely consumed beverage known for its health-promoting properties, is categorized into several varieties and exhibits potent antioxidant activity. Flavonoids isolated from white tea have demonstrated inhibitory effects against AGEs generated from BSA, fructose (Fru), and glucose [8]. Pu-erh tea has been shown to reduce AGE accumulation in glomeruli and confer protective effects against diabetes [9]. Theaflavin (TA), a major polyphenol in black tea, is formed through the oxidative coupling of catechin monomers and exhibits diverse bioactivities, including anticancer, anti-inflammatory, anti-obesity, and antidepressant effects [10]. Studies suggest that TA may have therapeutic potential for progressive neurodegenerative diseases and type 2 diabetes [11,12]. However, its effect on AGEs and the underlying mechanisms remain poorly understood.

The inhibitory effects of antioxidants on AGE formation can be further modulated by metal ion chelation. For instance, quercetin has been shown to inhibit glucose- and ribose-induced AGE formation by chelating magnesium, copper, or zinc ions [13]. Magnesium, an essential element involved in gene stability, DNA replication and repair, as well as in the reduction in oxidative stress and inflammation [14], has been reported to inhibit the generation of AGEs from human serum albumin (HSA) and glyoxal [15]. Similarly, manganese, another essential trace element that regulates bone growth, blood sugar, and the metabolism of carbohydrates and lipids [16], has been demonstrated to inhibit AGE formation in the BSA-glucose system [17]. Despite these findings, the combined effect of TA with essential metal ions (Mg^2+^ or Mn^2+^) on AGE inhibition remains unclear. Therefore, it is imperative to systematically evaluate the inhibitory effect of TA on AGE formation both in the presence and absence of these metal ions.

Multiple mechanisms can contribute to the inhibition of AGE formation, including scavenging free radicals, trapping reactive dicarbonyl compounds, chelating metal ions, and blocking glycation sites on proteins [18]. Elucidating these mechanisms requires a physiologically relevant model system. HSA, the most abundant plasma protein, consists of 585 amino acids organized into three domains (I-III), each containing two subdomains (A and B) [19]. It plays crucial roles in maintaining colloidal osmotic pressure, regulating blood pH, and transporting diverse ligands, making it a suitable model for glycation studies. Common reducing sugars involved in glycation reactions include glucose, Fru, lactose, and lactulose. Fru, the sweetest ketohexose and an isomer of glucose, is abundant in vegetables, fruits, and honey. Owing to its high sweetness, stability, low cost, and pronounced glycation activity, Fru is widely used in the food industry and glycation research [20]. Chaki et al. [21] established an AGE model by incubating HSA and Fru at pH 7.4 and 37 °C for 28 days. However, prolonged incubation at 37 °C is time-consuming and susceptible to microbial contamination. Given that previous studies have demonstrated that AGE formation at 50 °C shortens the incubation time by 7- to 15-fold without significant differences in intermediate product levels or AGE digestibility [22,23], the HSA-Fru model incubated at 50 °C was selected for this study to investigate the influence of metal ions on TA-mediated inhibition of AGEs. Furthermore, elucidating the interaction between HSA and TA is essential for clarifying the inhibitory mechanism. Our previous study demonstrated that blocking glycation sites contributes to the inhibition of AGE formation by caffeic acid (CA) and dihydrocaffeic acid, with CA exhibiting stronger inhibition due to its higher binding affinity for HSA [24]. Therefore, characterizing the interaction between HSA and TA, particularly in the presence of metal ions, is crucial for gaining mechanistic insights.

In summary, although the roles of certain metal ions in glycation have been partially elucidated, the effects of TA alone and in combination with Mg^2+^ or Mn^2+^, as well as the underlying mechanisms, remain unexplored. This study aimed to evaluate the inhibitory effect of TA on AGE formation and the interactions between TA and HSA, both in the presence and absence of Mg^2+^ or Mn^2+^, using spectroscopic techniques and molecular docking. Furthermore, the conformational changes in HSA following glycation and inhibition were monitored, and the antioxidant properties of TA were assessed. The findings provide theoretical insights into the influence of essential metal ions on the inhibition of AGEs by TA, which may contribute to the prevention and treatment of chronic diseases.

## 2. Results and Discussion

### 2.1. Effect of Mg^2+^ or Mn^2+^ on the Inhibition of AGEs by TA

#### 2.1.1. Fluorescence Spectra Study

**Figure 1:**
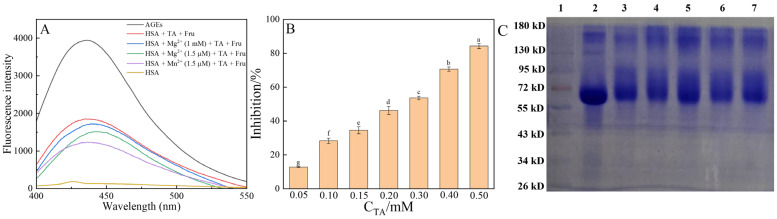
(**A**) Fluorescence spectra of native HSA, AGEs, HSA + TA + Fru, and HSA + Mg^2+^/Mn^2+^ + TA + Fru. (**B**) Inhibition percentages of AGE formation by various concentrations of TA. (**C**) SDS-PAGE analysis: (1) marker, (2) HSA, (3) AGEs, (4) HSA + TA + Fru, (5) HSA + Mn^2+^ (1.5 μM) + TA + Fru, (6) HSA + Mg^2+^ (1 mM) + TA + Fru, (7) HSA + Mg^2+^ (1.5 μM) + TA + Fru. The concentrations of HSA and Fru were 20 µM and 33 mM for panels (**A**,**B**), and 30 µM and 50 mM for panel (**C**), respectively. Different lowercase letters above the bars in panel (**B**) indicate statistically significant differences between groups (*p* < 0.05).

The inhibitory effect of TA on AGEs was evaluated after 8 days of incubation. As shown in Figure 1A, the fluorescence intensity of native HSA was very weak and negligible. A strong emission peak was observed at 440 nm, indicating the formation of AGEs. Upon the addition of TA, the fluorescence intensity decreased, suggesting that TA inhibits the formation of AGEs. A similar phenomenon has been reported for the inhibition of AGEs by rosmarinic acid [25]. As depicted in Figure 1B, the inhibitory effect of TA increased in a dose-dependent manner. The IC_50_ value of TA was calculated to be 0.22 mM, which is lower than that of aminoguanidine hydrochloride (a common AGE inhibitor, IC_50_ = 2.74 mM) or CA (IC_50_ = 0.26 mM). Furthermore, at a concentration of 0.3 mM, TA exhibited an inhibition rate of 52.89%, which was higher than that of aminoguanidine hydrochloride (11.32%) or CA (51.78%) [24]. These results indicate that TA has a stronger inhibitory effect on AGEs than AG and CA, demonstrating that TA is a potent AGE inhibitor. The superior inhibitory activity of TA compared to CA may be attributed to its stronger antioxidant capacity, which allows it to more effectively scavenge free radicals generated during the glycation process (details in Section 2.2.6).

The inhibition percentages in the presence of 1 mM Mg^2+^, 1.5 μM Mg^2+^, and 1.5 μM Mn^2+^ were 33.70%, 19.94%, and 42.57%, respectively, following the trend: 1.5 μM Mn^2+^ > 1 mM Mg^2+^ > 1.5 μM Mg^2+^. This trend suggests that Mg^2+^ and Mn^2+^ can inhibit the formation of AGEs, with Mn^2+^ exhibiting a more pronounced inhibitory effect than Mg^2+^. This may be attributed to metal ion-induced decomposition of AGEs or delayed conversion of dicarbonyl compounds to AGEs [26], as well as the strong antioxidant properties of Mn^2+^ [27]. As shown in Figure 1A, the fluorescence intensity of AGEs further decreased in the presence of both metal ions and TA. The inhibition percentages followed the order: 1.5 μM Mn^2+^ + TA (68.73%) > 1.5 μM Mg^2+^ + TA (61.50%) > 1 mM Mg^2+^ + TA (54.26%) > TA alone (52.89%). This trend is consistent with the binding affinity between TA and HSA in the absence and presence of metal ions (details in Section 2.2.2). These results suggest that both Mg^2+^ and Mn^2+^ enhance the binding of TA to HSA, thereby improving the inhibitory effect of TA on AGE formation. In addition, the inhibitory effect of the combination containing 1.5 μM Mg^2+^ and TA was more pronounced than that of 1 mM Mg^2+^ and TA, which contrasts with the trend observed for the metal ions alone. This result suggests that a higher concentration of Mg^2+^ may form a complex with TA or promote the interaction between Mg^2+^ and HSA, thereby reducing the effective binding of TA to HSA and diminishing its inhibitory effect on AGEs.

#### 2.1.2. Sodium Dodecyl Sulfate-Polyacrylamide Gel Electrophoresis (SDS-PAGE) Analysis

The formation of AGEs can induce protein cross-linking, leading to aggregation [28]. As shown in Figure 1C, the band corresponding to native HSA appeared at approximately 66 kDa, consistent with its theoretical molecular weight (66.5 kDa). The band for HSA incubated with Fru shifted upward, indicating the formation of high-molecular-weight AGEs. In the presence of TA, the intensity of the AGE band decreased, demonstrating that TA efficiently inhibits the formation of AGEs. The inhibitory effect of TA was further enhanced in the presence of metal ions. Notably, the band pattern of HSA + Mn^2+^ + TA + Fru closely resembled that of native HSA. According to Qin et al. [29], polyphenols can inhibit the aggregation of AGEs, thereby suppressing their formation. Consistent with this, the AGE band became fainter in the HSA + TA + Fru system, and this effect was more pronounced in the presence of metal ions. The improvement in inhibition followed the order: 1.5 μM Mn^2+^ + TA > 1.5 μM Mg^2+^ + TA > 1 mM Mg^2+^ + TA > TA alone, which is consistent with the fluorescence spectroscopy results.

#### 2.1.3. Scanning Electron Microscopy (SEM) Images

**Figure 2:**
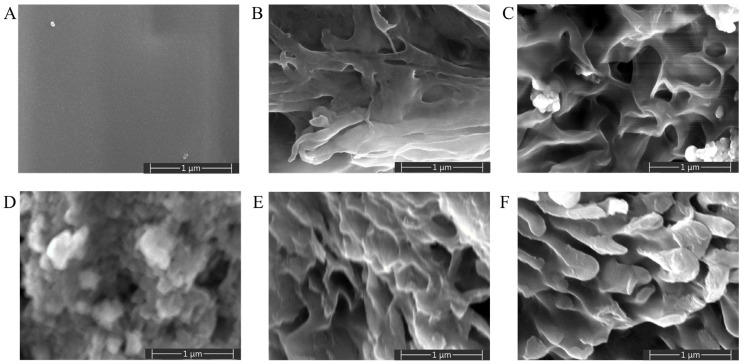
SEM images of (**A**) HSA, (**B**) AGEs, (**C**) HSA + TA + Fru, (**D**) HSA + Mg^2+^ (1 mM) + TA + Fru, (**E**) HSA + Mg^2+^ (1.5 μM) + TA + Fru, and (**F**) HSA + Mn^2+^ (1.5 μM) + TA + Fru.

The microstructures were examined to further confirm the inhibitory effect of TA on AGE formation in the presence and absence of Mg^2+^ or Mn^2+^. As shown in Figure 2, aggregates were observed in the Fru-induced sample compared to native HSA. In contrast, the aggregates became looser and more dispersed after the addition of TA, indicating the inhibitory effect of TA on AGEs. This observation is consistent with the reported ability of polyphenolic compounds to inhibit AGEs, as described by Han et al. [30]. Furthermore, the effect was more pronounced in the presence of metal ions. The degree of improvement followed the trend: 1.5 μM Mn^2+^ + TA > 1.5 μM Mg^2+^ + TA > 1 mM Mg^2+^ + TA > TA alone, which is consistent with the SDS-PAGE results.

#### 2.1.4. Analysis of Carbonyl and Thiol Contents

The carbonyl and thiol contents are associated with the redox status of the systems and can provide insights into protein conformational changes. As listed in Table 1, the carbonyl content of AGEs increased, while the thiol content decreased compared to native HSA. Upon the addition of TA, these values shifted toward those of native HSA. These findings indicate that certain amino acid residues and masked free thiol groups were oxidized during glycation, and TA can mitigate these changes as an effective AGE inhibitor. The results are consistent with those reported by Riaz et al. [31], in which baicalein inhibited the oxidation of HSA during glycation. Furthermore, the extent of mitigation in carbonyl and thiol contents followed the order: 1.5 μM Mn^2+^ + TA > 1.5 μM Mg^2+^ + TA > 1 mM Mg^2+^ + TA > TA alone. These results suggest that both Mg^2+^ and Mn^2+^ enhance the inhibitory effect of TA on AGEs, which is consistent with the findings from the preceding experiments.

### 2.2. Inhibitory Mechanisms of AGEs by TA in the Absence and Presence of Metal Ions

#### 2.2.1. Polarity Changes Determined by Fluorescence Spectra

**Figure 3:**
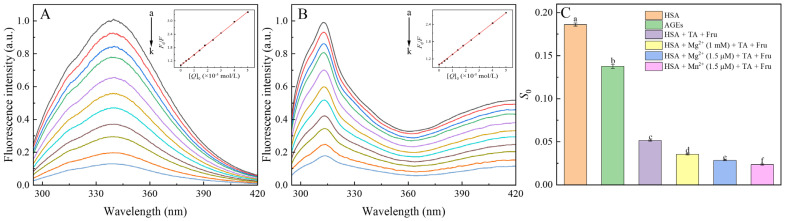
Fluorescence spectra of (**A**) HSA and (**B**) AGEs with increasing concentrations of TA at 298.2 K. *C*_HSA/AGEs_ = 2 μM, *C*_TA_ = 0–50 μM (a→k). (**C**) *S*_0_ values of ANS (40 μM) bound to HSA, AGEs, HSA + TA + Fru, or HSA + Mg^2+^/Mn^2+^ + TA + Fru. The concentration of HSA in (**C**) was 0–5 μM. Different lowercase letters above the bars for panel (**C**) indicate statistically significant differences between groups (*p* < 0.05).

Fluorescence spectroscopy provides a sensitive method for characterizing interactions between ligands and macromolecules. As shown in Figure 3 and Figure S1, HSA exhibited a strong emission peak at 338 nm. The addition of TA alone, or in combination with 1 mM Mg^2+^, 1.5 μM Mg^2+^, or 1.5 μM Mn^2+^, resulted in red shifts of 1 nm, 0.9 nm, 0.8 nm, and 0.6 nm, respectively. These shifts indicate that the chromophores of HSA are situated in a more hydrophilic environment upon binding with TA and metal ions. In contrast, the intrinsic fluorescence of AGEs exhibited a blue shift from 314 nm to 313.4 nm upon the addition of TA, indicating that the chromophores within AGEs reside in a more hydrophobic microenvironment.

Synchronous fluorescence spectroscopy was employed to further characterize environmental changes around tyrosine (Tyr) and tryptophan (Trp) residues. As presented in Figure S1, a decrease in fluorescence intensity was observed for both Tyr and Trp, accompanied by red shifts of 0.4 nm and 1 nm, respectively. These results confirm that the microenvironments of both residues become more hydrophilic, consistent with the findings from intrinsic fluorescence measurements. The more pronounced shift, observed for Trp compared to Tyr, and its closer resemblance to that of native HSA suggests that TA binds preferentially near the Trp residue of HSA. This interpretation is supported by the binding site analysis (Section 2.2.3) and docking results (Section 2.2.4).

The fluorescent probe 1-anilinonaphthalene-8-sulfonic acid (ANS) was employed to assess changes in surface hydrophobicity induced by glycation and ligand binding. As shown in Figure 3C, the *S*_0_ value of HSA decreased after glycation with Fru and decreased further upon the addition of TA. This decrease may be attributed to the occupation of hydrophobicity regions on HSA by Fru and TA or the introduction of polar groups from TA. A similar phenomenon was reported in our previous study on the inhibition of AGEs by CA [24]. However, the effect of TA was more pronounced than that of CA, likely due to structural differences: CA, with its more compact structure, predominantly binds within the internal pockets of HSA, whereas the larger structure of TA may perturb the surface properties of HSA. Additionally, the *S*_0_ value decreased further in the presence of metal ions, following the order: 1.5 μM Mn^2+^ + TA > 1.5 μM Mg^2+^ + TA > 1 mM Mg^2+^ + TA > TA alone. This trend suggests that both Mg^2+^ and Mn^2+^ promote the binding of TA to HSA and facilitate the influence of TA on the surface hydrophobicity of HSA, which is consistent with the binding affinities determined in the following section.

#### 2.2.2. Binding Properties of HSA + TA in the Absence and Presence of Mg^2+^ or Mn^2+^

As shown in Figure 3A and Figure S1, the fluorescence intensity of HSA decreased upon the addition of TA, both in the presence and absence of metal ions. The molar ratios of TA to HSA at half-quenching at 298.2 K were approximately 12:1, 10:1, 9:1, and 7:1 for the systems containing HSA + TA, HSA + 1 mM Mg^2+^ + TA, HSA + 1.5 μM Mg^2+^ + TA, and HSA + 1.5 μM Mn^2+^ + TA, respectively. These results indicate that both Mg^2+^ and Mn^2+^ enhance the binding between TA and HSA, with the enhancement effect following the order: 1.5 μM Mn^2+^ + TA > 1.5 μM Mg^2+^ + TA > 1 mM Mg^2+^ + TA > TA alone. As reported by Kaspchak et al. [32], Mg^2+^ or Mn^2+^ can bind to nonspecific negatively charged regions or imidazole groups via electrostatic forces or chelation, thereby promoting protein-ligand binding. Subsequent studies revealed that the primary driving force for TA binding to HSA is hydrophobic interaction, with electrostatic forces making a substantially smaller contribution. TA binds near HIS 247, which contains an imidazole group (details in Section 2.2.4). Furthermore, compared to 1.5 μM Mg^2+^, a higher concentration of Mg^2+^ (1 mM) may promote its binding to TA or HSA, thereby partially interfering with the interaction between TA and HSA. Consequently, the enhancing effect of Mn^2+^ was more pronounced than that of Mg^2+^, and a higher concentration of Mg^2+^ reduced the efficacy of potentiation. This trend is consistent with the inhibitory effects on AGE formation, suggesting that metal ions influence the inhibitory activity of TA by modulating its binding to HSA.

In general, fluorescence quenching mechanisms can be categorized as static or dynamic, which can be distinguished based on the quenching rate constants (*k*_q_) calculated using Equation (1) [33]:

(1) $$F_{0}/F=1+k_{q}\tau_{0}{\left[Q\right]}_{0}$$

where *F*_0_ and *F* represent the fluorescence intensities of HSA in the absence and presence of TA or the combination of TA and metal ions. [*Q*]_0_ denotes the concentration of TA, and *τ*_0_ is the fluorescence lifetime of HSA (6 × 10^−9^ s), as determined by time-resolved fluorescence measurements.

The Stern-Volmer plot exhibited a linear relationship, indicating the presence of a single quenching mechanism. All regression equations in Table S1 exhibited excellent linearity (*R*^2^ > 0.99). As summarized in Table 2, the calculated *k*_q_ values exceeded 2 × 10^10^ L·mol^−1^·s^−1^, which is indicative of static quenching. However, the *k*_q_ values increased with temperature, which is atypical for static quenching. To further elucidate the quenching mechanism, time-resolved fluorescence spectroscopy was employed to measure the lifetimes of HSA and HSA + TA. As shown in Figure S1F, the fluorescence decay curves of HSA and HSA + TA were nearly superimposable. The measured lifetimes were 6.0225 ns for HSA and 5.8338 ns for HSA + TA, respectively. These results confirm that TA quenches the fluorescence of HSA through a static quenching mechanism. A similar phenomenon has been reported in the systems of piceatannol/oxyresveratrol + soy protein isolate [34]. 

In the static quenching process, the binding constant (*K*_a_), the number of binding sites (*n*), the enthalpy change (Δ*H*^o^), the entropy change (Δ*S*^o^), and the Gibbs free energy change (Δ*G*^o^) can be determined using Equations (2) and (3) [35]:

(2) $$\log(F_{0}/F-1)=\log K_{a}+n\log[Q]$$

(3) $$\ln K_{a}=-\frac{∆G^{o}}{RT}=-\frac{∆H^{o}}{RT}+\frac{∆S^{o}}{R}$$

where [*Q*] represents the free concentration of TA and *R* is the gas constant (8.314 J·mol^−1^·K^−1^).

As listed in Table 2, the values of *n* were close to 1, indicating a single binding site between TA and HSA or AGEs. The binding between HSA and TA was strong, with *K*_a_ values on the order of 10^5^. Furthermore, the *K*_a_ values for the AGEs + TA system at both 298.2 and 310.2 K were smaller than those for the HSA + TA system, suggesting that Fru hindered the binding of TA to HSA. Therefore, TA can inhibit the formation of AGEs by competing with Fru for binding sites on HSA, a phenomenon also observed in the inhibition of AGE formation by CA [24]. Notably, the binding affinity of HSA for CA (8.23 × 10^5^ L·mol^−1^) was stronger than that for TA (2.70 × 10^5^ L·mol^−1^), which contrasts with their respective inhibition abilities. This discrepancy suggests that blocking glycation sites represents only one of the inhibitory mechanisms involved, and that other pathways may contribute more significantly to the overall inhibition. The weaker binding of TA to HSA compared to CA can be attributed to two factors: (1) the larger molecular structure of TA results in greater steric hindrance upon binding to HSA; (2) the hydrogen bonds formed between HSA and CA are shorter than those between HSA and TA (details in Section 2.2.4), indicating stronger hydrogen bonding interactions in the HSA + CA system. Furthermore, the addition of metal ions enhanced the binding between TA and HSA, with the extent of enhancement following the order: 1.5 μM Mn^2+^ + TA > 1.5 μM Mg^2+^ + TA > 1 mM Mg^2+^ + TA > TA alone. These results indicate that both Mg^2+^ and Mn^2+^ can improve the inhibitory effect of TA on AGEs by promoting the binding between TA and HSA, a conclusion consistent with the inhibition assay and half-quenching results. Notably, the *K*_a_ values remain of a comparable magnitude at 298.2 and 310.2 K, and their trends are consistent with the inhibitory results at 323.2 K. This suggests that the binding mode does not change within this temperature interval and that the interpretation of the inhibition mechanism remains valid.

The increase in *K*_a_ values with rising temperature indicates that heating facilitates the binding between TA and HSA, which is further supported by the positive value of Δ*H*^o^. The negative values of Δ*G*^o^ confirm that the binding process is spontaneous. Both positive Δ*H*^o^ and Δ*S*^o^ values suggest that the interaction is primarily driven by hydrophobic interactions, in agreement with the findings reported by Xu et al. [36]. The positive values of Δ*H*^o^ and Δ*S*^o^ can be attributed to the desolvation effect and the release of water molecules from the hydrophobic binding pocket of HSA.

**Figure 4:**
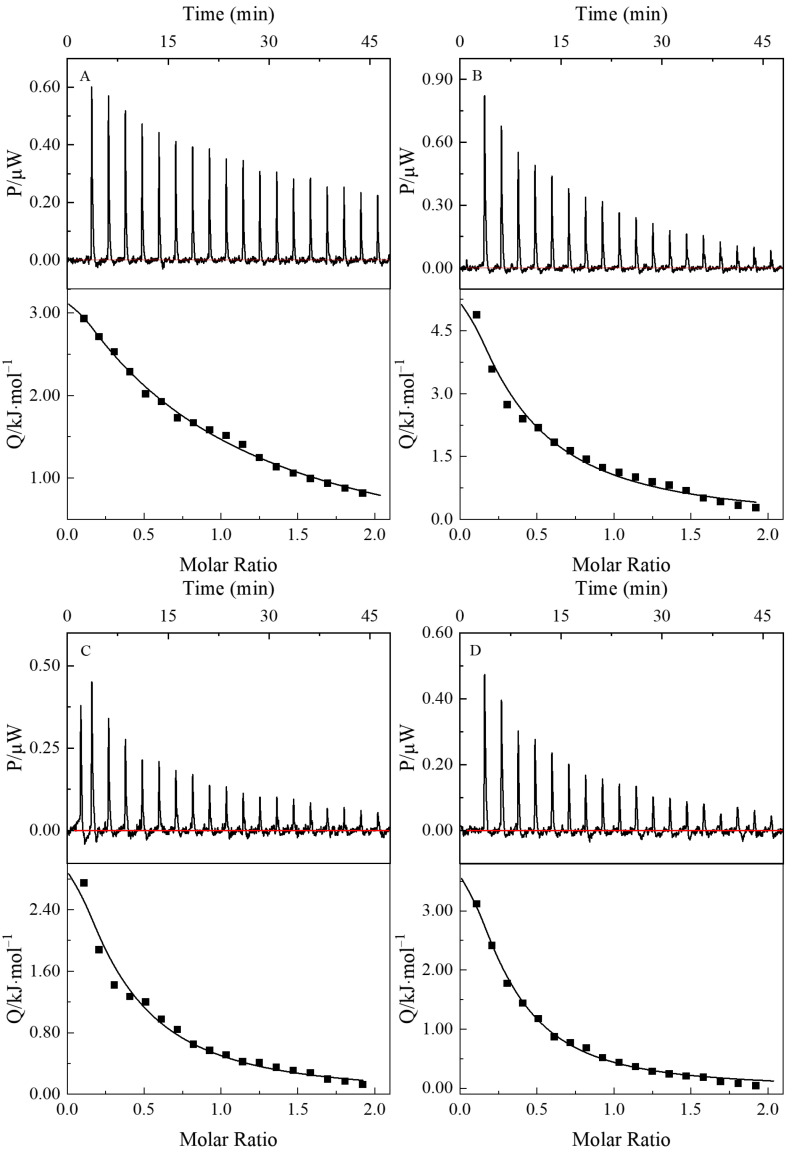
ITC titrations of TA (1 mM) into the cell containing (**A**) HSA (100 μM), (**B**) HSA + 1 mM Mg^2+^, (**C**) HSA + 1.5 μM Mg^2+^, and (**D**) HSA + 1.5 μM Mn^2+^ at 298.2 K.

To further support the above findings, isothermal titration calorimetry (ITC) experiments were conducted. As shown in Figure 4, the upper and lower panels show the heat flow for each titration over time and the net heat (after subtracting the dilution heat) plotted against the molar ratio, respectively. The interaction between TA and HSA in the presence or absence of Mg^2+^/Mn^2+^ is endothermic, and the one-set-of-sites model fitting demonstrates good agreement with the experimental data. As listed in Table 2, the *K*_a_ value for the HSA + TA system is on the order of 10^3^, which is lower than that obtained from fluorescence measurements. This discrepancy may be attributed to the assumption that Δ*H*^o^ in the van ’t Hoff equation remains constant within the temperature range used in the fluorescence experiments, which may not be completely consistent with the actual conditions [37]. The presence of metal ions facilitates the binding of TA to HSA and therefore improves the inhibitory effect of TA on AGE formation. The degree of enhancement follows the order: 1.5 μM Mn^2+^ + TA (7.4-fold) > 1.5 μM Mg^2+^ + TA (4.8-fold) > 1 mM Mg^2+^ + TA (3.7-fold) > TA alone (1.0-fold), which is consistent with the results obtained from fluorescence spectroscopy and inhibition assays. Moreover, both Δ*H*^o^ and Δ*S*^o^ were positive but decreased as the *K*_a_ values increased upon the addition of metal ions. These observations may be due to the combined effects of alterations in the dominant driving forces, conformational changes in the proteins, and desolvation upon complex formation.

#### 2.2.3. Determination of the Binding Sites

Warfarin (War) and ibuprofen (Ibu) were employed as site-specific fluorescent markers to identify the binding location of TA on HSA. As shown in Figure S2A, War exhibited relatively weak fluorescence intensity, which increased sharply upon binding to HSA. However, the fluorescence intensity decreased in the presence of TA, indicating that TA partially displaces War from its binding site. The addition of Mg^2+^ or Mn^2+^ showed only a slight effect. These results suggest that TA binds to the same site as War on HSA (site I). As shown in Figure S2B, Ibu displayed a strong emission peak, the intensity of which decreased upon the addition of HSA, confirming the binding between Ibu and HSA. The intensity of the Ibu + HSA system remained almost unchanged upon the addition of Mg^2+^ or Mn^2+^, indicating that metal ions have a negligible influence on the binding of Ibu to HSA. Furthermore, the intensity continued to decrease in the presence of TA, suggesting that TA does not compete with Ibu for its binding site (site II). Combining these findings, it is concluded that TA primarily binds to site I on HSA. This conclusion is further supported by the docking results presented in the following section. Moreover, metal ions also bind to site I but with a relatively weaker binding affinity compared to TA.

#### 2.2.4. Docking Analyses

**Figure 5:**
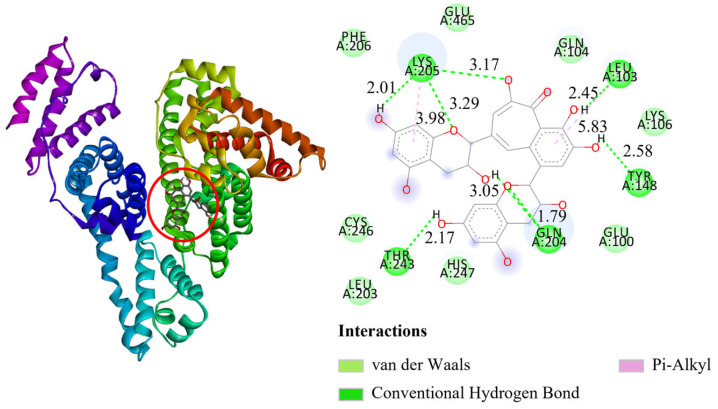
Molecular docking analysis of TA with HSA.

To ensure the reliability of the docking result, 100 independent docking runs were performed. The resulting conformations were clustered based on a root-mean-square deviation threshold of 2.0 Å. The largest cluster, comprising 32 out of 100 runs, was selected as the representative binding mode, indicating good statistical reproducibility. The average binding energy of this cluster was −42.86 kJ·mol^−1^, while the lowest energy among all runs was −45.24 kJ·mol^−1^, which occurred in only one run and was therefore not considered representative. Moreover, it should be noted that the docking-derived binding energy for the HSA + TA system is not comparable to the experimentally determined binding free energy of −31.00 kJ⋅mol^−1^. This discrepancy may be attributed to the structural differences between the X-ray crystal and the solvated protein in the experimental conditions [38]. The image corresponding to the lowest binding energy is presented in Figure 5. TA is bound within subdomain IIA of HSA (site I), consistent with the binding site identification results. The interaction between HSA and TA is primarily driven by hydrophobic interactions, with contributions from van der Waals forces and hydrogen bonding, as corroborated by thermodynamic studies.

TA interacts with LEU103 and LYS205 via hydrophobic interactions at distances of 5.83 and 3.98 Å. Additionally, hydrogen bonds are formed with LEU103, TYR148, GLN204 (O), GLN204 (H), LYS205 (H), LYS205 (O), LYS205 (O), and THR243, with bond distances of 2.45, 2.58, 1.79, 3.05, 2.01, 3.17, 3.29, and 2.17 Å, respectively. The relatively longer hydrogen bond distances in the HSA + TA system compared to those in the HSA + CA system may partially account for the weaker binding affinity of TA to HSA. It is noteworthy that, unlike CA, TA does not directly interact with the common glycation sites LYS525 or LYS199 [39]. However, since TA binds within subdomain IIA, it may sterically hinder the glycation of LYS199 by Fru, as LYS199 is situated at the entrance of this subdomain. Moreover, the larger molecular size of TA compared to CA may result in more pronounced steric hindrance, despite its relatively lower binding affinity for HSA. Of note, the docking was performed without the inclusion of metal ions, as its primary purpose was to identify the binding pocket of TA in HSA. Given the observed enhancement by Mg^2+^ or Mn^2+^, the detailed coordination and charge screening effects remain to be elucidated through future crystallographic or computational studies incorporating explicit metal parameterization.

#### 2.2.5. Conformational Changes Observation

**Figure 6:**
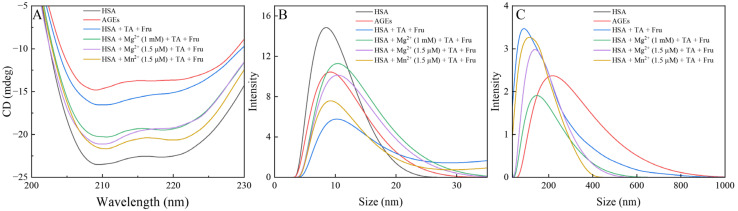
(**A**) CD spectra and (**B**,**C**) size distribution of native HSA, AGEs, HSA + Mg^2+^/Mn^2+^ + TA + Fru. The concentration of HSA in (**A**) and (**B**,**C**) was 2 μM and 15 μM, and the *x*-axis ranges of (**B**) and (**C**) were 0–35 and 35–1000 nm, respectively.

Circular dichroism (CD) spectroscopy was used to monitor conformational changes and characterize the secondary structures of proteins. As shown in Figure 6A, native HSA exhibited two characteristic negative peaks at 208 and 222 nm, typical of an α-helix structure [40]. The intensity of these peaks decreased following glycation with Fru, indicating that glycation alters the secondary structure of HSA. However, the spectral features were partially restored upon the addition of TA, suggesting that TA mitigates glycation-induced conformational changes. A similar phenomenon has been reported in studies on the inhibition of AGEs by sinensetin [41]. Moreover, the mitigating effect of TA was enhanced in the presence of metal ions. To quantify the conformational changes, the percentages of secondary structure contents were calculated and are summarized in Table 3. Glycation resulted in a decrease in α-helix content and an increase in β-sheet content, indicating structural unfolding and destabilization of HSA [42]. Notably, the α-helix content increased in the presence of TA compared to AGEs alone, suggesting that TA partially counteracts the structural disruption caused by glycation, although the conformation did not fully revert to that of native HSA. Moreover, the recovery of secondary structure was enhanced by metal ions, supporting their role in facilitating the inhibitory activity of TA. The extent of improvement followed the order: 1.5 μM Mn^2+^ + TA > 1.5 μM Mg^2+^ + TA > 1 mM Mg^2+^ + TA > TA alone. These results demonstrate that Mn^2+^ exerts the most pronounced effect, followed by 1.5 μM Mg^2+^ and 1 mM Mg^2+^, which is consistent with the trends observed in the inhibition assays.

Dynamic light scattering (DLS) was employed to investigate the conformational changes. As shown in Figure 6, native HSA exhibited a single sharp peak within the 0–35 nm range, consistent with its predominantly monomeric state. After glycation, the size distribution profile showed a pronounced, broadened aggregation tail that extended beyond 600 nm, indicating that HSA underwent aggregation during AGE formation. Notably, the addition of TA and metal ions markedly attenuated this long-tail aggregation, suggesting that they effectively mitigated the formation of aggregates induced by glycation. As listed in Table 3, the hydrodynamic diameters (*D*_h_) of HSA increased from 8.31 to 11.80 nm after glycation with Fru, implying an unfolding of the protein structure, which aligns with the CD results. Interestingly, upon the addition of TA, a further increase in *D*_h_ to 27.88 nm was observed. Although TA effectively prevented the aggregation tails during AGE formation, it resulted in the formation of moderately sized HSA-TA complexes, as indicated by a broadened peak centered around 100–200 nm. Because DLS reports an intensity-weighted average that is highly sensitive to larger species, the presence of these moderately sized complexes contributed disproportionately to the larger average *D*_h_ value. In contrast, the introduction of metal ions into the HSA + TA + Fru system resulted in a decrease in *D*_h_ compared to the system without metal ions. This phenomenon may arise from the structural alterations of HSA induced by chelation between TA and metal ions or by direct metal ion binding. Furthermore, the *D*_h_ values in the metal ion-containing systems followed the order: 1.5 µM Mn^2+^ > 1.5 µM Mg^2+^ > 1 mM Mg^2+^. This trend aligns with the results from AGE inhibition assays and binding affinity measurements.

#### 2.2.6. Antioxidant Activity Measurements

**Figure 7:**
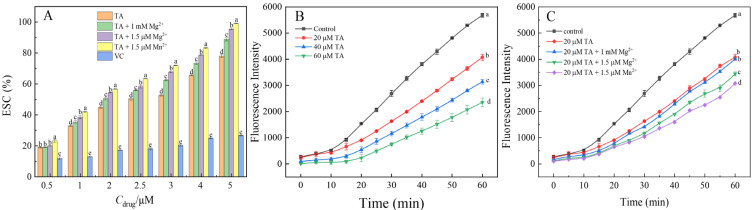
(**A**) ABTS^⋅+^ radical scavenging activity of TA, TA + Mg^2+^/Mn^2+^, and VC. (**B**) Inhibition of DCFH oxidation by TA in HepG2 cells, and (**C**) effect of Mg^2+^ and Mn^2+^ on this inhibition. The concentration of TA in (**A**), (**B**), and (**C**) was 0–5 μM, 0–60 μM, and 20 μM, respectively. The concentrations of VC, Mg^2+^, and Mn^2+^ were 0–5 μM, 1 mM/1.5 μM, and 1.5 μM, respectively. Different lowercase letters indicate statistically significant differences between groups (*p* < 0.05).

The antioxidant activity of TA was evaluated using an ABTS^⋅+^ radical scavenging assay, and the results are shown in Figure 7A. TA exhibited stronger scavenging activity than vitamin C (VC), indicating its potential as an effective antioxidant. The IC_50_ value of TA (2.02 μM) was lower than that of CA (8.07 μM) as reported previously [24], suggesting the superior antioxidant potency of TA. This difference may be attributed to the greater number of hydroxyl groups in TA compared to CA [43]. Furthermore, the antioxidant activity of the systems containing both TA and metal ions followed the order: 1.5 μM Mn^2+^ + TA > 1.5 μM Mg^2+^ + TA > 1 mM Mg^2+^ + TA > TA alone. This result indicates that both Mg^2+^ and Mn^2+^ enhance the antioxidant capacity of TA, with the most pronounced improvement observed in the presence of Mn^2+^, followed by 1.5 μM Mg^2+^ and 1 mM Mg^2+^. The IC_50_ values for the systems containing 1 mM Mg^2+^ + TA, 1.5 μM Mg^2+^ + TA, and 1.5 μM Mn^2+^ + TA were 1.70, 1.43, and 1.28 μM, respectively, further supporting this trend. The observed enhancement in antioxidant activity aligns with the results from the AGE inhibition assays and binding affinity measurements.

The cellular antioxidant activity (CAA) assay was utilized to evaluate the intracellular free radical scavenging capacity of TA. In this assay, the fluorescence intensity correlates positively with the amount of 2′,7′-dichlorofluorescein produced via the deacetylation and oxidation of 2′,7′-dichlorodihydrofluorescein diacetate (DCFH-DA). As shown in Figure 7B, the fluorescence intensity in both the control and TA-treated groups exhibited a slow increase during the initial 10 min, followed by a more rapid rise after this time point, exhibiting a clear inflection point. This can be interpreted as a kinetic competition between reactive oxygen species (ROS) generation and TA-mediated scavenging [44]. In the initial phase (0–10 min), ROS production was relatively low, and TA was sufficient to quench the generated ROS, resulting in a suppressed fluorescence signal. The inflection point at approximately 10 min likely reflects the threshold at which the rate of ROS generation begins to outpace the scavenging capacity of TA, leading to a net accumulation of ROS and a steeper increase in fluorescence thereafter. Notably, the fluorescence intensity in TA-treated samples remained lower than that of the control throughout the entire time course and decreased in a dose-dependent manner, confirming the protective effect of TA against ROS generation. The EC_50_ value of TA was 41.35 μM, which is lower than that of CA (87.09 μM) [24], indicating stronger intracellular antioxidant activity for TA. This result is consistent with the ABTS^⋅+^ free radical scavenging assay. Furthermore, the reduction in fluorescence intensity was more pronounced when TA was combined with metal ions (Figure 7C). The enhancement of antioxidant activity followed the order: 1.5 μM Mn^2+^ + TA > 1.5 μM Mg^2+^ + TA > 1 mM Mg^2+^ + TA ≅ TA alone. These results suggest that Mn^2+^ had the strongest promoting effect, followed by 1.5 μM Mg^2+^ and 1 mM Mg^2+^. This trend aligns with those observed in the inhibition of AGEs, binding affinity measurements, and ABTS^⋅+^ radical scavenging assays. In summary, TA inhibits AGE formation by both blocking glycation sites on HSA and scavenging the free radicals generated during glycation. Compared to CA, TA exhibited a stronger inhibitory effect on AGE formation, weaker binding affinity toward HSA, and greater antioxidant activity. Therefore, the antioxidant activity of TA likely contributes more significantly to its anti-AGE effect than does glycation site blocking.

## 3. Materials and Methods

### 3.1. Materials

Theaflavin (TA, mass fraction purity ≥ 95%), β-D-fructopyranose (Fru, ≥98%), human serum albumin (HSA, ≥96%), trichloroacetic acid (20%, *w*/*v*), 5,5′-dithiobis-(2-nitrobenzoic acid) (≥98%), 1-anilinonaphthalene-8-sulfonic acid (ANS, ≥98%), diammonium 2,2′-azino-bis(3-ethylbenzothiazoline-6-sulfonate) (ABTS, ≥98%), 2,2′-azobis(2-methylpropionamidine) dihydrochloride (AAPH, ≥98%), and vitamin C (VC, ≥99%) were acquired from Yuanye Biotechnology Co., Ltd. (Shanghai, China). Magnesium chloride hexahydrate (MgCl_2_⋅6H_2_O, ≥98%), 2,4-dinitrophenylhydrazine (DNPH, ≥98%), and human hepatocarcinoma HepG2 cells were obtained from Sangon Biotech Co., Ltd. (Shanghai, China), Macklin Biochemical Co., Ltd. (Shanghai, China), and Procell Life Science & Technology Co., Ltd. (Wuhan, China), respectively. Manganese (II) chloride tetrahydrate (MnCl_2_⋅4H_2_O, ≥99%) and 2′,7′-dichlorodihydrofluorescein diacetate (DCFH-DA, ≥97%) were purchased from Aladdin Biochemical Technology Co., Ltd. (Shanghai, China). Warfarin (War, ≥99%) and ibuprofen (Ibu, ≥99%) were sourced from J&K Chemical Ltd. (Beijing, China). All other chemicals were of analytical grade.

### 3.2. Apparatus

The fluorescence intensity and lifetime were measured using an F-7000 fluorescence spectrophotometer (Hitachi, Tokyo, Japan) and an FLS920 photoluminescence spectrometer (Edinburgh Instruments, Edinburgh, UK), respectively. The microstructure images were acquired using a Helios G4 CX dual-beam focused ion beam scanning electron microscope (Thermo Fisher Scientific, Waltham, MA, USA). The carbonyl and thiol contents, as well as ABTS^+^ radical scavenging activity, were evaluated using a U-3900H UV-vis spectrophotometer (Hitachi, Japan). The ITC experiments, CD spectra, DLS measurements, and CAA assays were performed using a MicroCal ITC200 titration calorimeter (GE Healthcare, Chicago, IL, USA), a J-810 spectropolarimeter (Jasco, Tokyo, Japan), a Zetasizer Nano ZS (Malvern Panalytical, Malvern, UK), and an Elx 808 microplate reader (Bio-Tek, Winooski, VT, USA), respectively.

### 3.3. Inhibitory Effect of TA on AGEs in the Presence and Absence of Mg^2+^ or Mn^2+^

#### 3.3.1. Inhibition of AGEs

AGEs were formed by incubating HSA and Fru according to a previously described method [24]. The groups were prepared in 0.2 M phosphate-buffered solution (PBS, pH 7.4) containing 0.02% ProClin300 as follows: (1) blank: HSA, (2) control: HSA + Fru (AGEs), (3) negative: HSA + TA or HSA + Mg^2+^/Mn^2+^ + TA, (4) positive: HSA + TA + Fru or HSA + Mg^2+^/Mn^2+^ + TA + Fru. The concentrations of HSA, Fru, TA, Mg^2+^, and Mn^2+^ were 0.3 mM, 0.5 M, 0–0.5 mM (0, 0.05, 0.1, 0.15, 0.2, 0.3, 0.4, 0.5 mM), 1 mM or 1.5 μM, and 1.5 μM, respectively. The non-cytotoxic concentrations of Mg^2+^ (1 mM) and Mn^2+^ (1.5 μM) were selected based on previous reports [45,46,47], with an additional Mg^2+^ concentration of 1.5 μM included for comparative purposes. After incubation at 50 °C for 8 days, the fluorescence intensities of all samples were recorded between 400 and 550 nm with an excitation wavelength of 370 nm. All samples were dialyzed against PBS (10 mM, pH 7.4) at 4 °C for 24 h to remove unbound Fru, then freeze-dried and stored at −20 °C. The inhibition percentage was calculated using Equation (4) [48]:

(4) $$\mathrm{Inhibition}\;(\%)=[(F_{c}-F_{b})-(F_{p}-F_{n})]/(F_{c}-F_{b})\times 100\%$$

where *F*_b_, *F*_c_, *F*_n_, and *F*_p_ represent the fluorescence intensities of the blank, control, negative, and positive groups, respectively. The half-maximal inhibitory concentration (IC_50_) values were determined using CompuSyn software (https://www.combosyn.com/feature.html, accessed on 16 March 2025).

A TA concentration of 0.3 mM, slightly above the IC_50_ value, was selected for subsequent experiments. The freeze-dried samples were redissolved in 10 mM PBS for further analysis.

#### 3.3.2. SDS-PAGE

SDS-PAGE was performed using a gel system consisting of 5% stacking gel and 10% separating gel. The procedure was conducted as follows: (1) HSA, AGEs, and lyophilized samples of HSA + TA + Fru or HSA + Mg^2+^/Mn^2+^ + TA + Fru (2 mg/mL) were mixed with 5× protein loading buffer at a 4:1 volume ratio and boiled for 5 min. (2) After centrifugation at 4000 rpm for 5 min, 10 μL of the supernatant was loaded into each well. The electrophoresis was initially carried out at 80 V for 0.5 h, followed by 120 V for 1 h. (3) The gels were stained with Coomassie Blue R250 fast-staining solution and destained in a solution containing 10% acetic acid and 45% methanol before image acquisition.

#### 3.3.3. SEM

The lyophilized samples of AGEs, HSA + TA + Fru or HSA + Mg^2+^/Mn^2+^ + TA + Fru, and native HSA were mounted on conductive adhesive plates. After removal of loose particles, the samples were sputter-coated with gold using a 10 keV acceleration voltage. All operations and imaging were performed using AutoScript 4 software (https://www.thermofisher.cn/cn/zh/home/electron-microscopy/products/software-em-3d-vis/autoscript-4-software.html, accessed on 14 October 2024).

#### 3.3.4. Determination of Carbonyl and Thiol Contents

The carbonyl content was quantified using DNPH with a molar extinction coefficient (*ε*) of 22,000 L⋅M^−1^⋅cm^−1^ [49]. Briefly, 1.2 mL of a 2 mg/mL solution from each sample (native HSA, AGEs, and lyophilized complexes of either HSA + TA + Fru or HSA + Mg^2+^/Mn^2+^ + TA + Fru) was mixed with 4.8 mL of DNPH (6 mM in 2.5 M HCl) under light-protected conditions for 1 h. The mixture was cooled on ice and precipitated by adding 6 mL of trichloroacetic acid. After centrifugation at 10,000× *g* and 4 °C for 10 min, the pellet was washed three times with 12 mL of a 1:1 (*v*/*v*) ethanol/ethyl acetate mixture. The final precipitate was dissolved in 3 mL of guanidine hydrochloride (6 M), and the absorbance was measured at 370 nm with appropriate background subtraction.

The free thiol contents of HSA, AGEs, and lyophilized HSA + TA + Fru or HSA + Mg^2+^/Mn^2+^ + TA + Fru were determined using Ellman’s method with *ε* = 13,600 L⋅M^−1^⋅cm^−1^ [50]. The samples (4 mg/mL, 3 mL) were incubated with or without 5,5′-dithiobis-(2-nitrobenzoic acid) (4 mg/mL, 50 μL) at 298.2 K for 0.5 h, and the absorbance was measured at 412 nm. The respective contents (*C*) of carbonyl and free thiol in the sample can be calculated using Equation (5) [49]:

(5) A=εCl

where *A* represents the absorbance at 370 or 412 nm; *l* is the optical path length (1 cm).

### 3.4. Inhibitory Mechanism of TA on AGEs in the Presence and Absence of Mg^2+^ or Mn^2+^

#### 3.4.1. Fluorescence Spectra

To explore the effect of Mg^2+^ or Mn^2+^ on the binding between TA and HSA, the binding assays were performed by maintaining the concentration of HSA or AGEs at 2 μM while varying the concentration of TA from 0 to 50 μM (0, 2, 4, 6, 10, 14, 18, 24, 30, 40, 50 μM). For the (HSA + Mg^2+^/Mn^2+^) + TA system, 2 μM HSA was pre-incubated with Mg^2+^ or Mn^2+^ (1 mM/1.5 μM or 1.5 μM) for 0.5 h, after which the same concentrations of TA were introduced. After incubation for 0.5 h at 298.2, 302.2, 306.2, and 310.2 K or at 298.2 and 310.2 K for AGEs + TA and (HSA + Mg^2+^/Mn^2+^) + TA systems, the fluorescence intensity was recorded between 295 and 420 nm with an excitation wavelength of 280 nm. The absorbance of all samples was measured to correct for the inner-filter effect using Equation (6) [51]:

(6) $$F_{\mathrm{corr}}=F_{\mathrm{obs}}\times e^{(A_{\mathrm{ex}}+A_{\mathrm{em}})/2}$$

where *F*_corr_ and *F*_obs_ represent the corrected and observed fluorescence intensities, and *A*_ex_ and *A*_em_ denote the absorbance at the excitation and emission wavelengths, respectively.

The synchronous fluorescence measurements were conducted using the same concentrations of HSA and TA as described above. After a 0.5 h incubation at 298.2 K, the spectra were recorded from 250 to 310 nm with wavelength intervals (Δλ) of 15 nm and 60 nm, corresponding to Tyr and Trp residues, respectively.

The fluorescence lifetimes were measured for the systems containing HSA (2 μM) and HSA + TA (6 μM). After incubation for 0.5 h at 298.2 K, the fluorescence lifetimes were obtained using the excitation and emission wavelengths of 280 and 338 nm, respectively. The data acquisition and fitting were performed using F900 software.

The surface hydrophobicity was assessed by mixing 1.99 mL of samples (HSA, AGEs, HSA + TA + Fru, or HSA + Mg^2+^/Mn^2+^ + TA + Fru at 0–5 μM) with 0.01 mL of ANS (40 μM), followed by incubation for 0.5 h at 298.2 K. The fluorescence intensities were measured from 400 to 600 nm with excitation at 390 nm. The surface hydrophobicity values (*S*_0_) were derived from the plots of fluorescence intensity versus protein concentration.

For the determination of binding sites, War and Ibu were used as site markers for sites I and II, respectively. The tested systems included War/Ibu alone, War/Ibu + HSA, (War/Ibu + HSA) + TA, and (War/Ibu + HSA) + Mg^2+^/Mn^2+^. The concentrations of War/Ibu, HSA, TA, and Mg^2+^/Mn^2+^ were 6 μM, 0.6 μM, 40 μM, and 1.5 μM, respectively. After the pre-incubation of War/Ibu with HSA for 0.5 h at 298.2 K, TA or metal ions were added and incubated for an additional 0.5 h. The fluorescence spectra were recorded in the ranges of 325–500 nm for War (excitation at 350 nm) and 270–420 nm for Ibu (excitation at 220 nm). The inner-filter effect was corrected for systems containing Ibu.

#### 3.4.2. ITC Experiments

All ITC experiments were performed at 298.2 K. The syringe was loaded with TA (1 mM), and the cell contained HSA (100 μM) in the absence or presence of Mg^2+^ (1 mM/1.5 μM) or Mn^2+^ (1.5 μM). The titration consisted of an initial 0.4 μL injection, followed by 18 subsequent 2 μL injections, with a reference power of 5 μcal/s. An interval of 150 s and a stirring speed of 750 rpm were applied to ensure baseline recovery and complete mixing. To correct for dilution and mixing effects, control titrations were carried out by injecting buffer into the HSA solution (with or without metal ions) and TA into buffer. All subtracted data were fitted using Origin 7.0 software supplied with the instrument.

#### 3.4.3. Docking Study

The three-dimensional structure of HSA (PDB ID: 1A06) was obtained from the Protein Data Bank (https://www.rcsb.org, accessed on 12 June 2025), and the structure of TA was sourced from the Automated Topology Builder (http://atb.uq.edu.au, accessed on 12 June 2025). The docking was performed using AutoDock 4.2.6 software with a grid map of 126 Å × 126 Å × 126 Å and a grid spacing of 0.375 Å. After 100 random runs, the resulting binding information was collected using AutoGrid 4.0 and VMD software (https://www.ks.uiuc.edu/Research/vmd/, accessed on 12 June 2025). To validate the docking reproducibility, 100 independent runs were performed, and the resulting poses were clustered using a root mean square deviation threshold of 2.0 Å.

#### 3.4.4. Conformational Changes

The CD spectra of HSA, AGEs, and lyophilized HSA + TA + Fru or HSA + Mg^2+^/Mn^2+^ + TA + Fru solutions (2 μM) were recorded from 200 to 230 nm after a 0.5 h incubation at 298.2 K, using a scanning speed of 200 nm⋅min^−1^. The secondary structure contents were analyzed using the DichroWeb server with the CONTIN algorithm.

The *D*_h_ values of HSA, AGEs, and lyophilized HSA + TA + Fru or HSA + Mg^2+^/Mn^2+^ + TA + Fru (15 μM) were measured after a 0.5 h incubation at 298.2 K and filtration through a 0.22 μm membrane.

#### 3.4.5. Antioxidant Activity Assays

The ABTS^⋅+^ solution was prepared by incubating a mixture of 2.5 mM ABTS and 1 mM AAPH in the dark for 40 min at 341.2 K. The solution was cooled to room temperature, filtered through a 0.22 μm membrane, and diluted to an absorbance of 0.76 ± 0.02 at 734 nm. To determine the effect of metal ions on the antioxidant activity of TA, 2.94 mL of ABTS^⋅+^ solution was mixed with TA, TA + Mg^2+^, TA + Mn^2+^, or VC. The concentrations of TA/VC, Mg^2+^, and Mn^2+^ were 0–5 μM, 1 mM/1.5 μM, and 1.5 μM, respectively. After 0.5 h of incubation at 298.2 K, the absorbance at 734 nm was measured. The scavenging activity was calculated using Equation (7) [52]:

(7) $$\mathrm{ESC}\left(\%\right)=\frac{\left(A_{0}-A_{1}\right)}{A_{0}}\times 100$$

where *A*_0_ and *A*_1_ represent the absorbance of the ABTS^⋅+^ solution in the absence and presence of samples, respectively.

For CAA assays, HepG2 cells were cultured in medium (90% DMEM, 9% fetal calf serum, and 1% penicillin) at 37 °C and 5% CO_2_, and seeded into a black 96-well plate at a density of 6 × 10^4^ cells per well. After 24 h, the medium was discarded, and the wells were rinsed with PBS. Subsequently, 50 μL of TA (0, 20, 40, and 60 μM) or TA + Mg^2+^ (1 mM/1.5 μM)/Mn^2+^ (1.5 μM), along with 50 μL of DCFH-DA (25 μM), were added to each well. The control wells received 100 μL of DCFH-DA alone. After a 1 h incubation and an additional PBS wash, 100 μL of AAPH (600 μM) was added to each well. The fluorescence intensity was measured every 5 min for 1 h with excitation and emission wavelengths of 485 and 538 nm, respectively. The CAA values were calculated using Equation (8) [53]:

(8) CAA value=(1−∫SA/∫CA)×100

where ∫SA and ∫CA represent the integrated areas under the fluorescence versus time curves for the sample and control, respectively. The half-maximal effective concentration (EC_50_) was determined from a plot of log(Fa/Fu) versus log(concentration), where Fa = CAA and Fu = 100−CAA.

### 3.5. Statistical Analysis

All experiments were performed in triplicate. The data were plotted using Origin 9.0 software, and statistical analyses were conducted using SPSS 19.0 with one-way analysis of variance (ANOVA). A *p*-value < 0.05 was considered statistically significant.

## 4. Conclusions

In this study, the inhibitory effect of TA on AGE formation and its underlying mechanisms, both in the absence and presence of Mg^2+^ or Mn^2+^, were systematically investigated using a combination of spectroscopic techniques and docking. The addition of metal ions significantly enhanced the inhibitory effect of TA on AGE formation, following the order: 1.5 μM Mn^2+^ + TA > 1.5 μM Mg^2+^ + TA > 1 mM Mg^2+^ + TA > TA alone. This enhancement may be attributed to the ability of Mg^2+^ and Mn^2+^ to promote both the binding of TA to HSA and the antioxidant activity of TA. Thermodynamic analysis indicated that the hydrophobic interactions were the major driving force for the binding process. Site marker experiments and docking results consistently identified site I (subdomain IIA) as the primary binding site of TA on HSA. Although TA does not bind directly to the key glycation residues such as LYS199, its binding within subdomain IIA sterically hinders the glycation of LYS199, which is situated at the entrance of this subdomain. Moreover, TA efficiently alleviated glycation-induced conformational changes in HSA and exhibited strong free radical scavenging capacity. These protective effects were further enhanced in the presence of metal ions. In comparison with CA, TA exhibited a stronger inhibitory effect on AGE formation and greater antioxidant activity, despite having a weaker binding affinity for HSA. These findings suggest that the potent antioxidant activity of TA plays a more critical role in inhibiting AGE formation than does the blocking of glycation sites. Other potential inhibitory mechanisms of TA against AGE formation, the quantification of AGEs in SEM images, and the biological responses to AGE samples warrant further investigation. Importantly, the enhancement effect of Mn^2+^ on TA-mediated AGE inhibition was confirmed to be reproducible at a physiological concentration (0.25 µM, Figure S3), and further investigation is warranted.

## Figures and Tables

**Table 1:** Carbonyl and thiol contents of HSA, AGEs, HSA + TA + Fru, and HSA + Mg^2+^/Mn^2+^ + TA + Fru.

	HSA *	AGEs *	HSA + TA + Fru	HSA + Mg^2+^ (1 mM) + TA + Fru	HSA + Mg^2+^ (1.5 μM) + TA + Fru	HSA + Mn^2+^ (1.5 μM) + TA + Fru
carbonyl content (µmol/g)	2.34 ± 0.03 ^f^	8.00 ± 0.04 ^a^	3.59 ± 0.02 ^b^	3.45 ± 0.01 ^c^	3.20 ± 0.01 ^d^	2.88 ± 0.01 ^e^
thiol content (µmol/g)	8.04 ± 0.09 ^a^	3.47 ± 0.03 ^f^	5.82 ± 0.02 ^e^	6.06 ± 0.02 ^d^	6.23 ± 0.01 ^c^	6.80 ± 0.03 ^b^

* Obtained from reference [24]. Different lowercase letters indicate statistically significant differences between groups (*p* < 0.05).

**Table 2:** Thermodynamic parameters for HSA + TA, AGEs + TA, and HSA + Mg^2+^/Mn^2+^ + TA at different temperatures.

	*T* (K)	HSA + TA	AGEs + TA	HSA + Mg^2+^ (1 mM) + TA	HSA + Mg^2+^ (1.5 μM) + TA	HSA + Mn^2+^ (1.5 μM) + TA
Fluorescence spectroscopy analysis						
*k*_q_ (10^12^ L·mol^−1^·s^−1^)	298.2	8.06 ± 0.03 ^j^	5.93 ± 0.03 ^l^	11.58 ± 0.07 ^d^	11.27 ± 0.05 ^e^	10.93 ± 0.03 ^f^
	302.2	8.32 ± 0.05 ^i^				
	306.2	9.08 ± 0.08 ^h^				
	310.2	9.93 ± 0.05 ^g^	6.41 ± 0.04 ^k^	32.17 ± 0.21 ^a^	18.99 ± 0.24 ^c^	22.58 ± 0.17 ^b^
*K*_a_ (10^5^ L·mol^−1^)	298.2	2.70 ± 0.05 ^j^	1.26 ± 0.02 ^l^	4.97 ± 0.02 ^f^	5.59 ± 0.04 ^e^	7.17 ± 0.05 ^b^
	302.2	2.93 ± 0.03 ^i^				
	306.2	3.16 ± 0.04 ^h^				
	310.2	3.34 ± 0.03 ^g^	1.58 ± 0.02 ^k^	5.77 ± 0.06 ^d^	6.49 ± 0.08 ^c^	7.83 ± 0.08 ^a^
*n* *	298.2	1.16 ± 0.01	1.12 ± 0.01	1.17 ± 0.01	1.20 ± 0.01	1.22 ± 0.02
	302.2	1.17 ± 0.01				
	306.2	1.16 ± 0.01				
	310.2	1.16 ± 0.01	1.14 ± 0.01	1.10 ± 0.02	1.16 ± 0.01	1.16 ± 0.02
Δ*G*^o^ (kJ·mol^−1^)	298.2	−31.00 ± 0.05 ^i^	−29.12 ± 0.04 ^j^	−32.52 ± 0.01 ^f^	−32.80 ± 0.02 ^e^	−33.42 ± 0.02 ^d^
	302.2	−31.62 ± 0.03 ^h^				
	306.2	−32.24 ± 0.03 ^g^				
	310.2	−32.79 ± 0.02 ^e^	−30.87 ± 0.03 ^i^	−34.21 ± 0.03 ^c^	−34.52 ± 0.03 ^b^	−35.00 ± 0.03 ^a^
Δ*H*^o^ (kJ·mol^−1^)		13.70 ± 0.58				
Δ*S*^o^ (J·mol^−1^·K^−1^)		149.95 ± 0.24				
ITC analysis						
*n* *	298.2	0.15 ± 0.01		0.16 ± 0.01	0.19 ± 0.01	0.21 ± 0.01
*K*_a_ (10^4^ L·mol^−1^)	298.2	0.43 ± 0.01 ^d^		1.56 ± 0.02 ^c^	2.02 ± 0.03 ^b^	3.16 ± 0.04 ^a^
Δ*G*^o^ (kJ·mol^−1^)	298.2	−20.71 ± 0.05 ^d^		−23.94 ± 0.03 ^c^	−24.58 ± 0.04 ^b^	−25.69 ± 0.03 ^a^
Δ*H*^o^ (kJ·mol^−1^)	298.2	53.16 ± 2.47 ^a^		25.45 ± 2.10 ^b^	10.41 ± 0.98 ^c^	9.03 ± 0.71 ^d^
Δ*S*^o^ (J·mol^−1^·K^−1^)	298.2	247.73 ± 8.28 ^a^		165.62 ± 7.05 ^b^	117.33 ± 3.28 ^c^	116.42 ± 2.40 ^c^

* No significant differences were found within this row (*p* > 0.05). Different lowercase letters indicate statistically significant differences between groups (*p* < 0.05).

**Table 3:** Secondary structure contents and *D*_h_ for HSA, AGEs, HSA + TA + Fru, and HSA + Mg^2+^/Mn^2+^ + TA + Fru.

System	CD				DLS
	α-Helix (%)	β-Sheet (%)	β-Turn (%)	Random Coil (%)	*D*_h_ (nm)
HSA *	50.50 ± 0.11 ^a^	8.60 ± 0.13 ^f^	15.40 ± 0.20 ^b^	25.50 ± 0.21 ^f^	8.31 ± 0.01 ^f^
AGEs *	37.20 ± 0.21 ^f^	17.80 ± 0.15 ^a^	17.50 ± 0.32 ^a^	27.50 ± 0.10 ^e^	11.80 ± 0.03 ^e^
HSA + TA + Fru	38.70 ± 0.15 ^e^	17.20 ± 0.05 ^b^	15.20 ± 0.40 ^b^	28.90 ± 0.05 ^d^	27.88 ± 0.03 ^a^
HSA + Mg^2+^ (1 mM) + TA + Fru	40.70 ± 0.15 ^d^	16.40 ± 0.20 ^c^	11.90 ± 0.05 ^c^	31.00 ± 0.01 ^b^	12.26 ± 0.01 ^d^
HSA + Mg^2+^ (1.5 μM) + TA + Fru	42.00 ± 0.05 ^c^	14.30 ± 0.20 ^d^	11.50 ± 0.05 ^d^	32.20 ± 0.05 ^a^	13.64 ± 0.02 ^c^
HSA + Mn^2+^ (1.5 μM) + TA + Fru	44.20 ± 0.10 ^b^	13.70 ± 0.10 ^e^	11.60 ± 0.10 ^d^	30.50 ± 0.03 ^c^	18.33 ± 0.02 ^b^

* Obtained from reference [24]. Different lowercase letters indicate statistically significant differences between groups (*p* < 0.05).

## Data Availability

The original contributions presented in this study are included in the article/Supplementary Materials. Further inquiries can be directed to the corresponding author.

## Supplementary Materials

The following supporting information can be downloaded at https://www.mdpi.com/article/10.3390/molecules31152648/s1, Figure S1: (**A**–**C**) Steady-state fluorescence spectra of HSA with TA in the presence of Mg^2+^ (1 mM), Mg^2+^ (1.5 μM), or Mn^2+^ (1.5 μM) at 298.2 K. (**D**,**E**) Synchronous fluorescence spectra of HSA with TA at Δλ = 15 and 60 nm at 298.2 K. *C*_HSA_ = 2 μM, *C*_TA_ = 0–50 μM (curves a→k). (**F**) Fluorescence decay curves of HSA (2 μM) in the absence and presence of TA (6 μM) at 298.2 K. The insets in (**A**–**C**) show the corresponding Stern-Volmer plots; Figure S2: Fluorescence spectra of (**A**) War and (**B**) Ibu in the absence and presence of HSA alone or HSA with TA, Mg^2+^, or Mn^2+^ at 298.2 K. The concentrations of War or Ibu, HSA, TA, and Mg^2+^ or Mn^2+^ were 6, 0.6, 40, and 1.5 μM, respectively; Figure S3: Fluorescence spectra of AGEs, HSA + TA + Fru, and HSA + Mn^2+^ + TA + Fru. The concentrations of HSA, Fru, and TA were 20 µM, 33 mM, and 20 µM, respectively; Table S1: Linear regression equations for fluorescence quenching of HSA + TA, AGEs + TA, and HSA + Mg^2+^/Mn^2+^ + TA at different temperatures.

## Abbreviations

The following abbreviations are used in this manuscript:

AGEs	advanced glycation end products
BSA	bovine serum albumin
Fru	fructose
TA	theaflavin
HSA	human serum albumin
CA	caffeic acid
Tyr	tyrosine
Trp	tryptophan
ANS	1-anilinonaphthalene-8-sulfonic acid
ITC	isothermal titration calorimetry
War	warfarin
Ibu	ibuprofen
CD	circular dichroism
DLS	dynamic light scattering
VC	vitamin C
CAA	cellular antioxidant activity
DCFH-DA	2′,7′-dichlorodihydrofluorescein diacetate
ROS	reactive oxygen species
DNPH	2,4-dinitrophenylhydrazine
ABTS	diammonium 2,2′-azino-bis(3-ethylbenzothiazoline-6-sulfonate)
AAPH	2,2′-azobis(2-methylpropionamidine) dihydrochloride
