# Supplementary material for: Unveiling the Role of Mg2+ and Mn2+ in Theaflavin-Mediated Inhibition of Advanced Glycation End Products Formation: Mechanistic Insights from Intermolecular Interaction
Source: Molecules. 2026 Jul 29;31(15):2648. doi: 10.3390/molecules31152648 (PMC13467637; doi:10.3390/molecules31152648)
Supplement: Supplementary file 1 [file molecules-31-02648-s001.zip › molecules-4412647-supplementary.pdf]

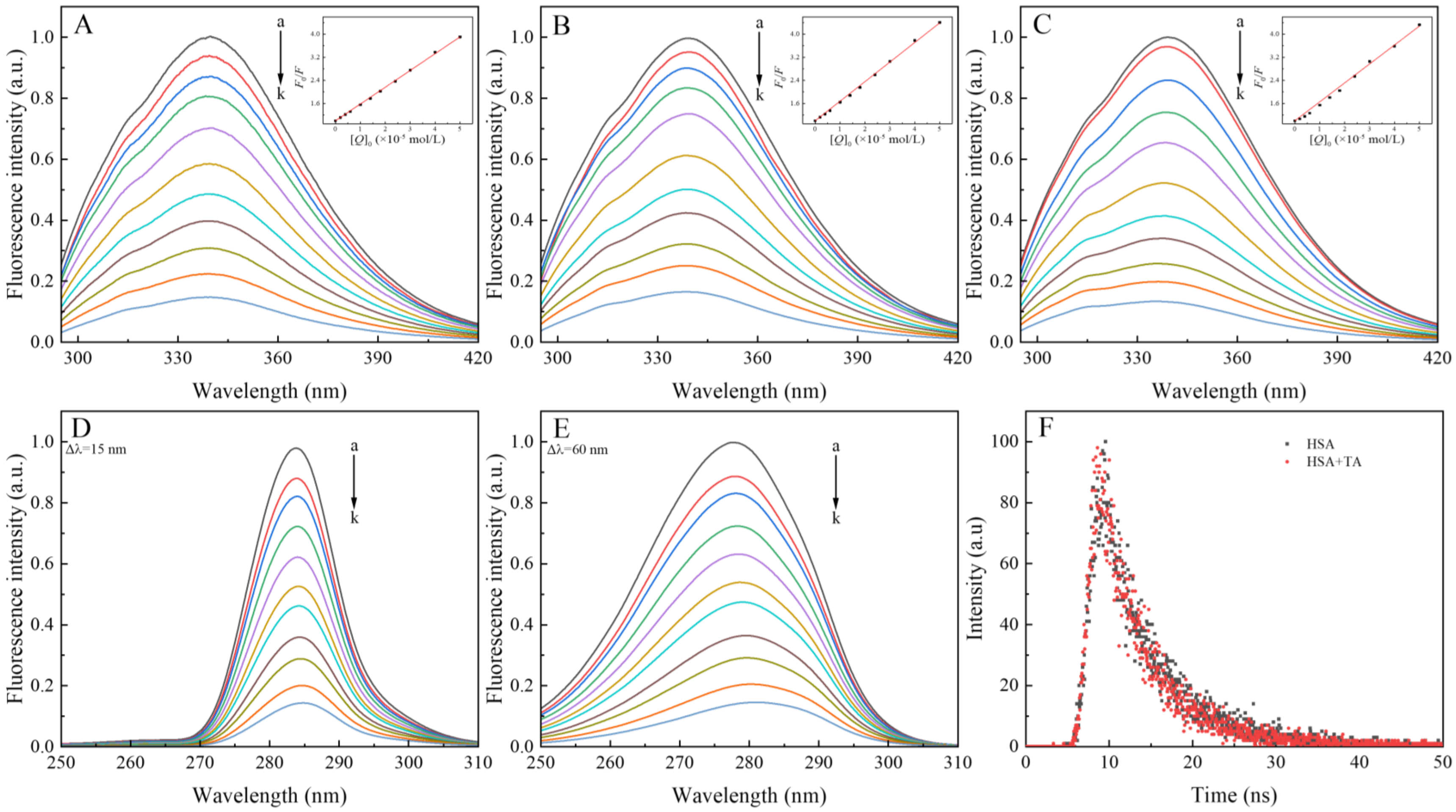

Figure S1. (A-C) Steady-state fluorescence spectra of HSA with TA in the presence of Mg^{2+} (1 mM), Mg^{2+} (1.5 μM), or Mn^{2+} (1.5 μM) at 298.2 K. (D, E) Synchronous fluorescence spectra of HSA with TA at Δλ = 15 and 60 nm at 298.2 K. C_{HSA} = 2 μM, C_{TA} = 0-50 μM (curves a→k). (F) Fluorescence decay curves of HSA (2 μM) in the absence and presence of TA (6 μM) at 298.2 K. The insets in (A-C) show the corresponding Stern-Volmer plots.

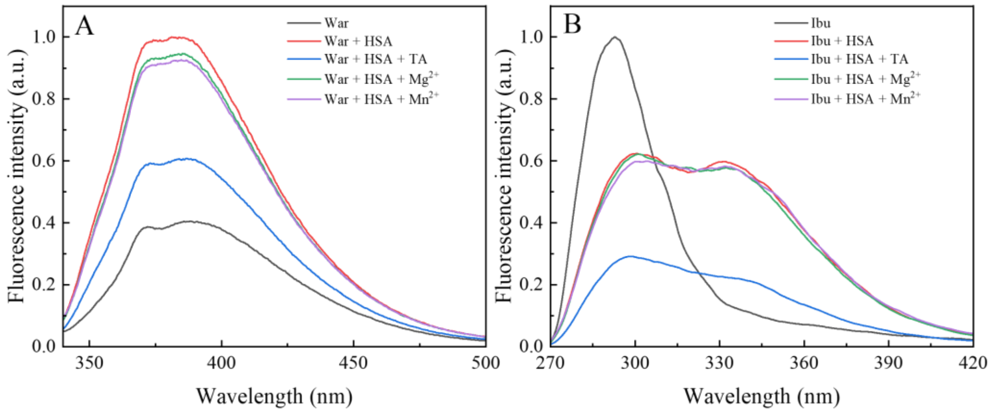

Figure S2. Fluorescence spectra of (A) War and (B) Ibu in the absence and presence of HSA alone or HSA with TA, Mg^{2+}, or Mn^{2+} at 298.2 K. The concentrations of War or Ibu, HSA, TA, and Mg^{2+} or Mn^{2+} were 6, 0.6, 40, and 1.5 μM, respectively.

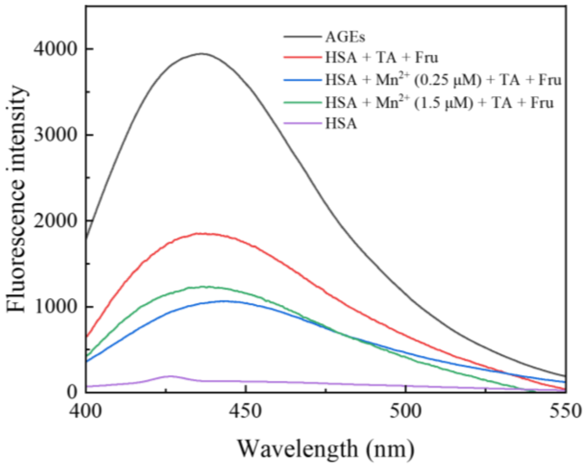

Figure S3. Fluorescence spectra of AGEs, HSA + TA + Fru, and HSA + Mn^{2+} + TA + Fru. The concentrations of HSA, Fru, and TA were 20 μM, 33 mM, and 20 μM, respectively.

Table S1. Linear regression equations for fluorescence quenching of HSA + TA, AGEs + TA, and HSA + Mg^{2+}/Mn^{2+} + TA at different temperatures.

	***T* (K)**	**HSA + TA**	**AGEs + TA**	**HSA + Mg^{2+} (1 mM) + TA**	**HSA + Mg^{2+} (1.5 μM) + TA**	**HSA + Mn^{2+} (1.5 μM) + TA**
	298.2	y = 48369x + 1	y = 35614x + 1	y = 69480x + 1	y = 67623x + 1	y = 65601x + 1
k_q	302.2	y = 49891x + 1				
$F_0/F = 1 + k_q\tau_0[Q]$	306.2	y = 54451x + 1				
	310.2	y = 59598x + 1	y = 38454x + 1	y = 193020x + 1	y = 113935x + 1	y = 135465x + 1
	298.2	y = 1.1607x +	y = 1.1210x + 5.1026	y = 1.1657x + 5.6964	y = 1.1990x + 5.7472	y = 1.2237x + 5.8557
n and K_a	302.2	5.4315 y = 1.1667x				
$\log(F_0/F - 1) = \log K_a + n\log[Q]$	306.2	+ 5.4662 y =				
	310.2	1.1597x + 5.5003 y	y = 1.1368x + 5.1978	y = 1.0983x + 5.7613	y = 1.1644x + 5.8122	y = 1.1643x + 5.8940
ΔH^o and ΔS^o		= 1.1611x + 5.5232				
$\ln K_a = -\frac{\Delta G^0}{RT} = -\frac{\Delta H^0}{RT} + \frac{\Delta S^0}{R}$	298.2	y = 1647.4x + 18.036				

All regression equations showed excellent linearity, with correlation coefficients (R^2) exceeding 0.99.
